# Reticulospinal Systems for Tuning Motor Commands

**DOI:** 10.3389/fncir.2018.00030

**Published:** 2018-04-18

**Authors:** Robert M. Brownstone, Jeremy W. Chopek

**Affiliations:** Sobell Department of Motor Neuroscience and Movement Disorders, Institute of Neurology, University College London London, United Kingdom

**Keywords:** locomotion, mesencephalic locomotor region, reticular formation, microcircuits, sleep atonia

## Abstract

The pontomedullary reticular formation (RF) is a key site responsible for integrating descending instructions to execute particular movements. The indiscrete nature of this region has led not only to some inconsistencies in nomenclature, but also to difficulties in understanding its role in the control of movement. In this review article, we first discuss nomenclature of the RF, and then examine the reticulospinal motor command system through evolution. These command neurons have direct monosynaptic connections with spinal interneurons and motoneurons. We next review their roles in postural adjustments, walking and sleep atonia, discussing their roles in movement activation or inhibition. We propose that knowledge of the internal organization of the RF is necessary to understand how the nervous system tunes motor commands, and that this knowledge will underlie strategies for motor functional recovery following neurological injuries or diseases.

## Introduction

As movement is necessary for the expression of all behavior, much of the vertebrate nervous system is involved in its production. A key site for integration of descending instructions to move is the reticular formation (RF), which is situated in the brain stem and comprised of multiple nuclei. The indiscrete nature of these regions combined with disparate neuronal types within each nucleus has led not only to some inconsistencies in nomenclature, but also to difficulties in understanding RF control of movement.

The RF is comprised of different neural types including monoaminergic, cholinergic, GABA/glycinergic and glutamatergic neurons, with glutamatergic reticulospinal neurons (RSNs) forming the key descending output. Axons of these neurons extend into the spinal cord such that RSN activity can lead to a variety of motor behaviors. Inputs to RSNs descend from diverse brain regions including the telencephalon, diencephalon and cerebellum, and ascend from the spinal cord. Local circuits within RF nuclei may also contribute to their output. RSNs therefore play a role in integrating and processing these diverse inputs in order to produce effective motor behaviors. Recent reviews have addressed the role of the RF in context specific locomotion (Kim et al., 2017) and the interaction of the control of posture and locomotion (Takakusaki et al., 2016). In this review, we will discuss glutamatergic RSN systems with a focus on their organization, connectivity with spinal neurons, control of hind limb movement, and the role of these systems in recovery of function after neurological injury. Additionally, we address the lack of information regarding the internal organization of RF nuclei and why this knowledge is vital for understanding how RS output is refined.

## Reticular Formation Nomenclature

One difficulty in determining specific functions of the RF arises from the non-discrete boundaries between nuclei and the inconsistent nomenclature in the literature. In this review, we will use the terminology as set out by Paxinos and Franklin (2008). As different investigators have used different terms, in this section we have attempted to harmonize the nomenclature through assessment of the available anatomical sections provided in publications focussed on the role of RSNs in movement.

The RF extends through the mesencephalon, pons and medulla and is traditionally divided into three columns—median, medial and lateral. In this review, we will focus on RSNs involved in limb movement, which arise from the medial column in the pons and medulla. Descending neurons from the median column arise from the raphe nuclei and are responsible for serotoninergic neuromodulation (see review: Schmidt and Jordan, 2000). The neurons of the lateral column and those in the mesencephalic RF do not project to the spinal cord.

The medial pontine RF, from rostral to caudal, comprises the nucleus reticularis pontis oralis (PnO), from the decussation of the superior cerebellar peduncles to the trigeminal motor pool, and the nucleus reticularis pontis caudalis (PnC), overlapping with PnO and extending caudally to the facial nucleus. Situated dorsal to these nuclei are the pontine tegmental nuclei, divided into ventral (region referred to as the ventral tegmental field, or VTF), dorsal (region referred to as the dorsal tegmental field, or DTF), and lateral tegmental nuclei, as well as the sublaterodorsal tegmental (SLDT) nucleus. The dorsal tegmental nucleus extends caudally to lie dorsal to the gigantocellular reticular nucleus (GRN, *vide infra*), by which point the ventral and lateral tegmental nuclei merge to form the laterodorsal tegmental nucleus (LDT). In addition, rodents may have a ventral nucleus of the medial pontine RF (PnV, Liang et al., 2011) that contains a small population of RS neurons, although it has been suggested this is part of the rostral medullary GRN (see Sivertsen et al., 2016).

The rostral portion of the medial medullary RF (medRF) begins at the most caudal portion of the PnC, with the GRN lying ventral to PnC and extending from the facial nucleus to the obex. The GRN (sometimes referred to as Gi) is a large nucleus that contains the largest cells in the RF. Ventral to this is the pars alpha of the GRN (GiA), which merges with the Gi pars ventral (GiV) caudally. In the cat literature, the GRN is often referred to as the nucleus reticularis gigantocellularis (NRGc) or gigantocellular tegmental field (FTG), and the GiA/GiV as the nucleus reticularis magnocellularis (NRMc) or magnocellular tegmental field (FTM, see Noga et al., 1988; Takakusaki et al., 2016), with medullary sections 9 mm caudal to the junction of the superior and inferior colliculi (P9) in the cat corresponding approximately to Bregma −7 in the mouse (Paxinos and Franklin, 2008). The dorsal paragigantocellular nucleus (DPGi) is dorsal to the GRN, and the parvocellular reticular nucleus (PCRt) is dorsolateral to the GRN. The intermediate reticular zone (IRt) which contains the nucleus ambiguus, separates the GRN from the PCRt (Paxinos et al., 2012). The lateral paragigantocellular nucleus (LPGi), lies lateral to GiV and ventromedial to nucleus ambiguus. In the caudal medulla which lacks giant GRN neurons, the central nucleus can be subdivided into the medRF ventral part (MdV), and more cellular dorsal part (MdD), separated by the caudal IRt and located in the caudal medulla lateral to the medial longitudinal fasciculus.

It should be noted that the borders between these regions are somewhat indistinct, necessitating a degree of caution when interpreting experimental findings involving either electrical stimulation (Mori et al., 1978; Garcia-Rill and Skinner, 1987; Takakusaki et al., 2016) or local injections (Noga et al., 1988; Takakusaki et al., 2016; Capelli et al., 2017). The comparison of histological sections between studies is helpful, but co-labeling with antibodies against choline acetyltransferase would be helpful in aligning sections based on consistent cholinergic nuclei such as motor pools.

## Reticulospinal Neurons Are Command Neurons for Movement

### RSNs Are Evolutionary Conserved Command Neurons

The reticulospinal (RS) system is a distributed network of neurons extending from the caudal midbrain through the pons and medulla (Peterson, 1984). RSNs receive inputs from rostral motor centers and have axons that descend through the ventrolateral funiculus of the spinal cord to form synapses with spinal interneurons and motoneurons that participate in movement. As RSNs are located between higher centers that select movement and spinal cord circuits where movement is organized, RSNs may be considered as command neurons.

Command neurons are widespread across invertebrate and vertebrate species. To be classified as a command neuron, a candidate neuron must satisfy the criteria of both sufficiency and necessity for initiating a given behavior (Kupfermann and Weiss, 1978). Command neurons in the brain stem have been identified in a number of vertebrate species in which they have been shown to be involved in motor behaviors such as escape and locomotion.

Dating back more than 500 million years, the early appearing agnathans, lampreys and hagfish, developed RS command neurons called Müller cells, that have axons that cross in the brainstem and descend the length of the spinal cord to evoke swimming (Shapovalov, 1972). Perhaps the prototypical RS command neuron, though, is the Mauthner cell, initially described in teleosts which arose ~310 million years ago. Given that Mauthner cells were readily identifiable and that they share similar location, morphology, and synaptic connectivity across species, they have been well studied as command neurons (Sillar et al., 2016). Mauthner cells are likely present in lower vertebrates such as lamprey, but their function has mostly been studied in later-evolving fish as well as amphibians.

Two Mauthner cells, located directly opposite each other near the midline of the medulla can readily be identified in the RF based on their large cell size and location at the level of the 4th rhombomere (Sillar et al., 2016; Hildebrand et al., 2017). The axons of Mauthner cells cross in the brain stem and project through the spinal cord where they form glutamatergic, excitatory connections to large primary motoneurons and premotor excitatory interneurons (Fetcho and Faber, 1988; Faber et al., 1989; Fetcho, 1991). These interneurons have descending projections, are electrotonically coupled to large motoneurons, and form chemical synapses with small motoneurons (Fetcho, 1992). The Mauthner cell axon is also electrotonically coupled to glycinergic commissural interneurons that inhibit the large motoneurons and interneurons on the contralateral side (Yasargil and Sandri, 1990; Fetcho, 1991).

Mauthner cells receive multiple sensory inputs (Sillar et al., 2016). In response to acoustic stimuli (cf. startle response in humans below), Mauthner cells fire a single action potential (Zottoli, 1977). Based on the connectivity described above, this results in excitation of contralateral motoneurons and inhibition of ipsilateral motoneurons. This produces a fast and forceful C-start escape, arising from the initial C-bend, in which the fish moves away from the initial stimulus (Fetcho, 1992). Thus, Mauthner cell activation produces a conserved stereotypical C-start response that mediates escape (Eaton et al., 1988; Faber et al., 1989).

It is now recognized that the C-start escape response is not necessarily stereotypical, and that depending on the stimulus site and intensity, combinations of RSNs, including Mauthner cells, can produce variations of the escape response (Eaton et al., 2001). For example, it was recently demonstrated that Mauthner cells are involved in a second type of escape response, the S-start escape, when bilaterally activated (Liu and Hale, 2017). The selection of the S- vs. C-start was determined by the recruitment of segmental inhibitory neurons in spinal circuits, which would modify the response. The adaptability in behavioral responses mediated by the RS system indicates that responses to command neuron activity are variable and state-dependent.

With further study, it became apparent that the Mauthner cell has multiple smaller homologoues and that they are organized in a segmental plan, similar to the segmented organization seen in invertebrates (Kimmel, 1993). It was also demonstrated that there are additional command neurons in the teleost RS system, which comprises approximately 500 neurons (Prasada Rao et al., 1987). Thus, even in these relatively early appearing teleosts, complex descending systems were present.

As supraspinal systems developed to control limbed movement, the RS system remained a dominant system mediating movement. Across species including frog, turtle, rat, cat and rhesus monkey, RSNs project to and monosynaptically excite limb motoneurons (Shapovalov, 1972). It is noteworthy that, in addition to the RS system, other descending systems such as the vestibulospinal system appeared beginning in amphibians (Shapovalov, 1972). While these other systems evolved further in reptiles, the RS system also expanded and diversified to include, for example, inhibitory RSNs. With further evolution of corticospinal including corticomotoneuronal systems in primates, the RS system persisted, producing responses in spinal motoneurons similar to those seen in fish. Thus, the RS system remains conserved though evolution (Shapovalov, 1972), and plays an important role in vertebrate, including human, movement.

In fact, the RS system was not only conserved but it flourished in evolution. For instance, compared to the 500 RSNs in the fish, there are approximately 50,000 RSNs in the mouse. Of these, approximately 19,000 are located in the GRN alone (Liang et al., 2011). Based on a comparison of EPSPs and IPSPs across species, Shapovalov concluded “the basic similarity between reticulo-motoneuronal projections across all vertebrates investigated may evidently be attributable to the common origin of the reticulospinal system, which undergoes mainly quantitative changes in the course of evolution” (Shapovalov, 1972, p. 353). In other words, as behavior became more complex, so too did the RS system. But the fundamental architecture and role of the RS system persisted across species: RSNs are large cells with dense arborizations and large fast-conducting axons that descend in the spinal cord, forming synapses with interneurons and motoneurons in multiple segments in order to produce movement.

### Reticulospinal Neurons Project to Spinal Interneurons and Motoneurons

The influence of the brainstem on posture was documented by Sherrington (1898) who noted that decerebration led to “decerebrate rigidity” or hyperactivity of extensor muscles, suggesting that there is an excitatory influence from sub-cerebral structures to the spinal cord. In the 1940s Rhines and Magoun demonstrated that following decerebration, stimulation of the ventral medulla could result in the complete loss of muscle tone, and cessation of stimulation led to the immediate return of hyperextensor activity, demonstrating that the RS system had both an excitatory and inhibitory influence on postural muscles (Magoun and Rhines, 1946; Rhines and Magoun, 1946). They also demonstrated that the RS system could either facilitate or inhibit spinal reflexes in the cat (Magoun, 1944; Magoun and Rhines, 1946; Rhines and Magoun, 1946), and found that the area responsible for inhibition resided in the ventral rostral medulla (Magoun and Rhines, 1946), whereas the excitatory regions extended through the pons and medulla (Rhines and Magoun, 1946). However, Sprague and Chamber (1954) found that in intact cats, for the most part, micro-stimulation of the RF led to reciprocal (flexion-extension as well as right-left) responses in the limbs rather than generalized facilitation or inhibition. Neurons in the caudal pons and GRN form either excitatory (Grillner and Lund, 1968) or inhibitory (Llinas and Terzuolo, 1964a, b) connections with flexor or extensor hindlimb motoneurons. In addition to the hindlimb responses, RS stimulation can produce direct excitation or inhibition of neck, back, and forelimb motoneurons (Wilson and Yoshida, 1969; Wilson et al., 1970; Peterson et al., 1978). In non-human primates, activation of movement by RS stimulation is achieved by both direct and indirect connectivity to motoneurons (Riddle et al., 2009). Thus, RSNs produce effects that are heterogeneous and widespread in the spinal cord.

It is important to note that, in contrast to other descending systems such as the lateral vestibulospinal and corticofugal systems, the RS system is not exclusively excitatory. A proportion of RSNs descending in the MLF are inhibitory (Du Beau et al., 2012), and approximately 20% of RS synaptic contacts on propriospinal and spinal commissural interneurons in the lumbar spinal cord are inhibitory (Mitchell et al., 2016). Recently it has been shown that glycinergic neurons located in the GRN descend to the lumbar spinal cord (Valencia Garcia et al., 2018; *vide infra*).

Within the RF, specific regions have been found to be responsible for specific responses (Drew and Rossignol, 1990). Ipsilateral projecting pontine RSNs outnumber contralateral projecting pontine RSNs three to one and are located throughout the pontine RF whereas the contralateral projecting neurons are concentrated in the rostral pontine RF (Sivertsen et al., 2016). In neonatal mice, medial neurons in the medRF predominantly activate lateral motor column (limb-innervating) MNs in the lumbar spinal cord, whereas lateral medRF neurons predominantly activate medial motor column (axial muscle-innervating) MNs (Szokol et al., 2008). In both cases, these actions may be mediated through polysynaptic pathways mediated by ipsilateral and contralateral descending spinal commissural interneurons (Szokol et al., 2011; Perreault and Glover, 2013).

Microstimulation within the RF can result in movement across multiple joints or limbs. For example, stimulation that produced forelimb movement was regularly accompanied by movement of the head or hindlimbs (Drew and Rossignol, 1990). Furthermore, homologous to Mauthner cell responses, motoneuron responses were not restricted to one side: microstimulation could evoke ipsilateral flexion and contralateral extension of the either the fore or hind limbs (Drew and Rossignol, 1990). These evoked movements are consistent with the axonal projections of RSNs, with 85% of axons extending to both the cervical and lumbar enlargements, and, at least in the cervical enlargement, axons projecting bilaterally (Peterson et al., 1975). Thus, RSNs have divergent spinal projections and are involved in multi-joint, multi-limb motor commands. In summary, the RS system in the mammal is functionally organized and acts to coordinate multiple movements including excitation and inhibition across joints and across limbs.

### medRF Reticulospinal Neurons and Locomotion

The rhythm and pattern of locomotion are produced by spinal cord circuits in response to descending commands (Graham Brown, 1911; Jankowska et al., 1967). RF locomotor command neurons are activated by an upstream center called the mesencephalic locomotor region (MLR). MLR stimulation produces locomotion in decerebrate cats (Shik et al., 1966), rats (Skinner and Garcia-Rill, 1984), and mice (Roseberry et al., 2016; Stecina, 2017; Caggiano et al., 2018) through a pathway mediated by medRF RSNs (Noga et al., 2003). The medRF has been shown to be crucial for locomotion in a number of other species as well, including lamprey (McClellan and Grillner, 1984), ducks and geese (Steeves et al., 1987) and guinea pigs (Marlinskii and Voitenko, 1992). In the cat, medRF RSNs in the GRN are necessary for MLR-evoked locomotion, and are sufficient to induce locomotor activity through electrical (Jordan et al., 2008) or chemical stimulation (Noga et al., 1988). These “locomotor” medRF RSNs also receive input from the contralateral cerebellar locomotor region (Mori et al., 1998) and from the ipsilateral lateral hypothalamic area, termed the subthalamic locomotor region (Sinnamon and Stopford, 1987). Given the multitude of locomotor-related inputs, Orlovskii (1970) stated that “the invariable mediating link for initiating locomotion is the reticulospinal system.”

Locomotor medRF RSNs are fast conducting (Orlovskii, 1970; Degtyarenko et al., 1998; Noga et al., 2003) and glutamatergic (Douglas et al., 1993; Jordan et al., 2008). They are phasically active during spontaneous locomotion in thalamic cats (Shimamura et al., 1982; Shimamura and Kogure, 1983), treadmill locomotion in unrestrained cats (Drew et al., 1986; Matsuyama and Drew, 2000), and spontaneous or MLR-evoked fictive locomotion in the absence of movement related feedback in decerebrate cats (Perreault et al., 1993). Locomotion-inducing RSN axons descend in the ventrolateral funiculus (Steeves and Jordan, 1980), may innervate the ipsi- or contra-lateral spinal cord (Matsuyama et al., 1988, 1999), and are known to innervate commissural interneurons in the lumbar spinal cord (Matsuyama et al., 2004; Jankowska, 2008). That is, RSNs in the medRF GRN provide extensive innervation to bilateral spinal motor regions, and seemingly fulfil the criteria of command neurons for locomotion.

While RSNs thus integrate inputs from multiple higher centers in order to send commands to the spinal cord, there may also be local processing of commands within the RF. Neurons within the RF have heterogeneous characteristics, leading to the suggestion that there may be local circuit processing of reticulospinal commands (Shimamura et al., 1980), but this has not been explored in detail. For example, a subset of neurons in this region in lamprey (Einum and Buchanan, 2004; Buchanan, 2011) and cat (Shimamura et al., 1980, 1982) receive inputs from the spinal cord. In the cat, it has been suggested that the phasic activity of RSNs during fictive locomotion may arise from inputs from the spinal cord (Perreault et al., 1993). This hypothesis is supported by the demonstrated phase-dependent modulation of RSN activity in response to cutaneous stimulation during locomotion (Drew et al., 1996). How these ascending inputs are processed and how they might affect the descending commands is not yet clear.

There is also evidence that some RSNs receive neuromodulatory input (Takakusaki et al., 1993a), indicating that their activity may be state dependent (Takakusaki et al., 2016). While there has been focus on modulation of RSNs by acetylcholine (Takakusaki et al., 1993b; Le Ray et al., 2010), serotonin also may play a role (Takakusaki et al., 1993a). Given the importance of serotoninergic activity in locomotion (Schmidt and Jordan, 2000), it is possible that this modulatory system is involved in both brain stem and spinal circuits for locomotion.

Pathways for different types of locomotor activity, such as escape vs. exploration, may differ (Sinnamon, 1993; Jordan, 1998). The MLR is comprised primarily of the cuneiform nucleus (Jordan, 1998), but recent evidence in the mouse suggests that while higher speed locomotion is mediated by the cuneiform nucleus, exploratory activity is initiated by a region just ventral to this, the pedunculopontine nucleus (PPN, Caggiano et al., 2018). The PPN has diverse projections to RF nuclei (Gi, GiV, GiA, LPGi, MdV), whereas the cuneiform projections are more restricted (LPGi, GiV, GiA but not the Gi or MdV, Caggiano et al., 2018). While cat studies have pointed towards the medial regions of the medRF as the command for locomotion (Noga et al., 2003), there has been a recent suggestion that in the mouse, LPGi activation is necessary for higher speed locomotion (Capelli et al., 2017). This suggests that there may be species differences in descending commands, a difference in the state of the circuits being studied across experiments, or that locomotor activity is initiated via indirect pathways in some experiments (see Noga et al., 2003). In summary, it is clear across multiple species that the medRF plays a key role in integrating and processing instructions for locomotion from more rostral centers.

In addition to their well-defined role in initiating locomotion, RSNs have recently been implicated in stopping locomotion. Optogenetic activation of a subset of glutamatergic neurons defined by expression of the transcription factor Chx10 located in the PnC and GRN regions can lead to cessation of locomotor activity in the mouse (Bouvier et al., 2015) and lamprey (Juvin et al., 2016). Three activity patterns in RSNs were seen in this region during swimming in lamprey: those that fired exclusively at the onset of swimming (“start” RSNs), those that fired throughout the swimming bout (“start and maintain” RSNs), and those that fired prior to the termination of locomotion (“stop” RSNs; Juvin et al., 2016). This indicates that there is a diversity of neuronal function within the RSN. In the mouse and possibly lamprey, the behavioral response from the stop RSNs is likely mediated by spinal inhibitory neurons targeted by these descending glutamatergic RSNs (Bouvier et al., 2015; Juvin et al., 2016).

The ability of RSNs in the medRF/GRN to act as command neurons for a variety of behaviors in addition to starting and stopping locomotion is highlighted by its involvement in a multitude of tasks ranging from, for example, maintaining posture during walking to atonia during sleep (*vide infra*).

### Interaction of Reticulospinal Systems for Locomotion and Posture

In the late 1970s, it was shown that postural adjustments associated with locomotion originated in the caudal portion of the pontine RF (see Mori, 1987). Stimulation of the dorsal area of the caudal pontine tegmental field (DTF) resulted in the relaxation of extensor muscles, whereas stimulation of the ventral area of the caudal pontine tegmental field (VTF) resulted in activation or an increase in extensor muscle tone. More recently, it has been shown that injections of cholinergic and serotonergic agonists into the PnO resulted in atonia and hypertonia respectively. These effects were mediated through medRF RSNs, with atonia-related RSNs located in the dorsomedial part of the medRF and hypertonus-related RSNs located in the ventromedial part of the medRF (Takakusaki et al., 2016). Thus it is clear that RSNs, through excitation and inhibition of spinal circuits, regulate muscle tone (for review see Takakusaki et al., 2016).

Extensor muscle tone is necessary for locomotion as there must be adequate force production to support the body and propel limbs forward (Mori et al., 1978, 1982). The interplay of posture and locomotion was demonstrated during MLR evoked locomotion, when stimulation of the DTF halted locomotion and stimulation of the VTF resulted in enhanced locomotor activity or a change in gait pattern (Mori et al., 1978). In freely moving cats with electrodes implanted in the DTF or VTF and the MLR, DTF stimulation during walking (naturally or MLR induced) led to sequential postural reduction to a sitting then prone position, and VTF stimulation when lying led to a rise to stance followed by locomotion (Mori et al., 1986).

The co-expression of postural tone and locomotion is likely due to shared neural pathways. For example, in addition to projecting to locomotor neurons in the GRN, the MLR also projects to postural areas (DTF and VTF). About 70% of DTF neurons are activated by MLR stimulation, with most being tonically active but some demonstrating rhythmic activity (Kawahara et al., 1985). Thus the MLR through two DTF pathways may participate in both tonic regulation of posture for locomotion and phasic regulation contributing to step cycle timing.

### Reticulospinal Neurons and Reaching

RSNs are involved in movements other than posture and locomotion. For example, some RSNs in the cat are active during the preparatory phase in anticipation of reaching, while others are active during the actual reaching task (Schepens, 2004). Anatomical evidence using viral tracing has demonstrated glutamatergic RSNs in the MdV that form synapses exclusively with forelimb and not hindlimb MNs, unlike other neurons, including those in the GRN, which form synapses with both forelimbs and hindlimb MNs (Esposito et al., 2014). Furthermore, these synapses are motor pool specific, appearing on biceps but not triceps motoneurons. Inhibition of these RSNs resulted in decreased ability of the mice to accurately reach and grasp during a food pellet challenge. Because these RSNs are not involved in hindlimb movement, we will not consider them further here.

### Reticulospinal Neurons and Sleep Atonia

While descending commands can stop locomotion (*vide supra*), the prototypical “stop” command is that which produces sleep atonia, in which limb muscle tone is curtailed to prevent movement during REM sleep (Saper et al., 2010). This atonic command originates in the SLDT nucleus, which contains glutamatergic neurons that project both to GRN and to the spinal cord (Sastre et al., 1981; Chase et al., 1986; Saper et al., 2010). Both of these targets contribute to sleep atonia, but whether the direct descending command from the GRN is inhibitory and/or whether its inhibitory effects are mediated by spinal interneurons is not clear (Fuller et al., 2007; Arrigoni et al., 2016). Recent evidence, however, suggests that there is descending inhibitory input that produces sleep atonia (Valencia Garcia et al., 2018).

In cats, lesions to the subcoeruleus region (analogous to the SLDT) result in behavior in which the cats appear to act out their dreams during sleep (Mouret et al., 1967; Henley and Morrison, 1974). Similarly, lesions to the SLDT in rodents led to complex motor behaviors during REM sleep (Lu et al., 2006). These behaviors are akin to REM behavior disorder (RBD) in humans (Schenck and Mahowald, 1995). That RBD can be localized to the SLDT has been shown in a case report of a woman with a lesion in this region who suffered from RBD and somnambulism (Limousin et al., 2009). Interestingly, RSNs that are associated with specific movements during waking are also active during REM sleep (Siegel et al., 1981). This suggests that extraneous movements during RBD are produced by the same neurons as during wakefulness. RBD is perhaps an extreme example of pathological RSN circuit selection. Other examples could include other disorders affecting movement selection such as dystonic syndromes.

## Intrinsic Organization of the Reticular Formation

Clearly, rostral centers must activate different populations of RSNs in order to produce selected movements. Given that there can be net inhibitory (sleep atonia) and net excitatory (locomotion) effects produced by RSNs, we propose that movement selection circuits activate smaller ensembles or microcircuits of RSNs in order to facilitate specific movements whilst inhibiting other movements. This concept is not unlike that of the direct and indirect pathways of the basal ganglia that cooperate in movement selection (Cui et al., 2013). This example also demonstrates that the control of moving and not moving are intimately intertwined, and that neurological disorders that alter either one or the other of these two classes of microcircuits can lead to significant impairment in quality of life. How these microcircuits interact with each other, however, is yet to be defined.

What determines the tuning of appropriate commands? As RSNs are involved in diverse movements and comprise a subset of RF neurons, it would be prudent to ask whether RSNs simply integrate and relay inputs from higher brain centers to the spinal cord, or whether local microcircuits participate in refining motor commands.

Whilst our knowledge of the functional and topographical organization of RSNs is increasing, little is known about the internal organization of the RF. Some neurons within these nuclei are inhibitory, presumably interneurons (Sivertsen et al., 2016), but there is little information about local connectivity. Several modeling studies have addressed this lack of understanding of the internal circuitry. One proposal is that the RF is a “small-world network” in which dense connectivity within small groups of nodes is responsible for appropriate action selection (Humphries et al., 2006). Individual nodes would comprise small diameter local interneurons, most likely inhibitory, that project medially and laterally and connect with larger projecting cells (likely RSNs) within their own node. Node to node connectivity would be via axon collaterals of the large projecting neurons which would form synapses at relatively short distances, allowing for nearby nodes to activate quickly and concurrently (Humphries et al., 2007).

Experimental data to support this model are in short supply to date. But it is clear that knowledge of the internal organization of the RF is critical to understand how the RF and RSNs are selected to produce a wide array of finely tuned motor commands.

## Reticulospinal Neurons in Humans and Their Role in Recovery of Motor Function

The corticofugal system plays an important role in human movement (Lemon, 2008). Axon collaterals from descending corticospinal neurons have diffuse projections, including to the RF (Kita and Kita, 2012). It is of interest that, although there was a deficit in fractionated finger movements in monkeys following lesions of these tracts below the level of the collaterals to the RF, other movements including climbing and running persisted (Lawrence and Kuypers, 1968a, b). Furthermore, monosynaptic corticomotoneuronal synapses are not necessary for hand movements in non-human primates, which improved over time following pyramidal tract lesions (Sasaki et al., 2004). Similarly, rare reports of sectioning these tracts in humans have demonstrated initial hemiparesis followed by recovery, ultimately leaving patients with a relative lack of movement deficit (Bucy et al., 1964). These data suggest that other systems such as RSNs can play a prominent role in movement in primates, including humans.

RS systems have been studied in humans via the startle reflex. This reflex response is seen across species and been shown to originate in the caudal brainstem—most likely the PnC (Hammond, 1973; Leitner et al., 1980; Davis et al., 1982). The startle reflex is seen in response to a loud acoustic stimulus (cf. Mauthner cells, above), and is stereotypically comprised of activation of the sternocleidomastoid muscle, followed by cranial nerve innervated muscles and rectus abdominus, and finally forelimb and hindlimb muscles (Rothwell, 2006). Evidence for a brainstem origin for this response has come from electrophysiological studies. When the instruction to start voluntary movement was combined with a loud auditory cue, the time to movement onset was significantly reduced (Rothwell, 2006). For example, the onset to volitional arm movement was found to be 150 ms, whereas arm movement initiated by the startle response can occur in 80 ms. This reduced latency has also been associated with an increased rate of force development, as well as increased force output (Anzak et al., 2011). Moreover, in more complex movements involving sequential activation of agonist and antagonist muscles, auditory stimulation sped up the activation of movement without affecting the pattern of muscle activity (Valls-Solé et al., 1999). Together, these data all point towards a subcortical, and more specifically RS, origin for startle reflexes.

The startle response in humans integrates with voluntary movements and may thus facilitate a potential therapeutic approach for regaining volitional movement post neurological insults to the cortex such as stroke (Rothwell, 2006) or to enhance motor performance in diseases such as Parkinson’s disease (Anzak et al., 2016). In stroke patients, when the startle response was combined with voluntary movement, the latency to EMG response was shorter and the response was much larger than when either was performed alone, leading to the suggestion that detail of voluntary movement can be stored in the brainstem and can be accessed to produce movement in the absence of descending inputs from the cerebral cortex (Rothwell, 2006).

Given that RSNs are important in movement, and that many neurological injuries occur above the level of the RF, it is reasonable to ask whether the RS system could be a therapeutic target for therapies aimed at recovery of motor function in neurological diseases and injuries such as stroke (Baker et al., 2015). Data from non-human primates supports this concept (Zaaimi et al., 2012). Presumably, intentional signals from higher centers would still need to reach the RF, but as noted above, there are many supramedullary inputs that converge on RSNs. For example, following unilateral stroke, there are non-crossed cortico-reticular inputs that could possibly harness the unaffected ipsilateral cortex to promote RF plasticity and motor functional recovery (Jankowska and Edgley, 2006). The RS system is well connected to play a key role in recovery from motor deficits, as it projects to all levels of the spinal cord including to key movement circuits.

Such RS-mediated functional recovery need not be limited to axial movement. Although initially proposed to be important for axial and proximal muscles, it is becoming clear from studies in non-human primates that RSNs are also involved in hand function (Riddle et al., 2009), and could thus be a substrate for functional recovery of hand movements following neurological injury (Baker, 2011). In support of this possibility, recent evidence from humans points to the RS system as being involved in gross hand function after spinal cord injury (Baker and Perez, 2017).

To promote RSN-mediated recovery of motor function, however, several challenges exist. It will be necessary to understand the functional organization of motor command circuits in the RF, including how nodes or microcircuits in the RF are engaged to produce movement, whether individual microcircuits participate in more than one motor behavior, how individual microcircuits are recruited by rostral circuits, whether these circuits are under neuromodulatory control, and whether “on” movement and “off” movement microcircuits are simultaneously recruited to refine motor commands. Understanding these concepts could form the foundation for future therapeutic strategies.

Ultimately, it is clear that our focus in understanding the RF should include not only an “integrate and relay” function, but also an understanding of the internal organization and processing that controls RSNs such that a great versatility of movements can be produced. This understanding will lead not only to improved knowledge of how we move, but potentially also to new experimental paradigms to understand motor system changes in injury and disease, and ultimately to the development of new therapeutic strategies aimed at improving motor functional recovery.

## Author Contributions

RB and JC conceived and wrote the manuscript.

## Conflict of Interest Statement

The authors declare that the research was conducted in the absence of any commercial or financial relationships that could be construed as a potential conflict of interest.

## Abbreviations

DTF: dorsal tegmental field
Gi: gigantocellular reticular nucleus
GiA: gigantocellular reticular nucleus, pars alpha
GRN: gigantocellular reticular nucleus
LPGi: lateral paragigantocellular nucleus
MdV: medulla reticular formation-ventral part
medRF: medulla reticular formation
MLR: mesencephalic locomotor region
PnC: pontine reticular formation, caudal part
PnO: pontine reticular formation, oral part
PPN: pedunculopontine nucleus
RF: reticular formation
RSN: reticulospinal neurons
SLDT: sublaterodorsal tegmental
VTF: ventral tegmental field.
